# Modeling of the fracture energy on the finite element simulation in Ti6Al4V alloy machining

**DOI:** 10.1038/s41598-021-98041-5

**Published:** 2021-09-16

**Authors:** Carolina Bermudo Gamboa, Tobias Andersson, Daniel Svensson, Francisco Javier Trujillo Vilches, Sergio Martín-Béjar, Lorenzo Sevilla Hurtado

**Affiliations:** 1 Department of Civil, Material and Manufacturing Engineering, EII, University of Malaga, C/Dr. Ortiz Ramos s/n, 29071 Malaga, Spain; 2 School of Engineering Science, Högskolan i Skövde, University of Skövde, Högskolevägen, 541 28 Skövde, Sweden

**Keywords:** Mechanical engineering, Characterization and analytical techniques, Computational science

## Abstract

One of the main problems that exists when working with Finite Element Methods (FEM) applied to machining processes is the lack of adequate experimental data for simulating the material properties. Moreover, for damage models based on fracture energy, the correct selection of the energy value is critical for the chip formation process. It is usually difficult to obtain the fracture energy values and requires complex tests. In this work, an analysis of the influence of this fracture energy on the cutting force and the chip generation process has been carried out for different sets of cutting parameters. The aim is to present an empirical relationship, that allows selecting the fracture energy based on the cutting force and cutting parameters. The work is based on a FEM model of an orthogonal turning process for Ti6Al4V alloy using Abaqus/Explicit and the fracture energy empirical relation. This work shows that it is necessary to adjust the fracture energy for each combination of cutting conditions, to be able to fit the experimental results. The cutting force and the chip geometry are analyzed, showing how the developed model adapts to the experimental results. It shows that as the cutting speed and the feed increase, the fracture energy value that best adapts to the model decreases. The evolution shows a more pronounced decrease related to the feed increment and high cutting speed.

## Introduction

Titanium alloys are important for the engineering field and are nowadays common in the aerospace, aeronautical, automotive, and biomedical industries. This is due to their combination of excellent mechanical and physical–chemical properties. Among the different titanium alloys, the Ti6Al4V alloy is the one widely used inairframe structure manufacture^1^. It presents a very good strength-to-weight ratio and superior corrosion resistance^2,3^. However, this alloy also gives rise to high machining costs due to its low thermal conductivity, high chemical reactivity and its ability to maintain hardness at high temperatures, leading to fast degradation of the cutting tool^4–6^. These problems are increased due to the current trend to reduce or eliminate the use of metalworking fluids (MWFs) when machining is performed^7^ due to social-economic and environmental aspects^8^. Dry machining reduces environmental pollution and health risks. However, a faster tool wear and surface integrity degradation is apparent in dry machining^9^.

The low machinability of the Titanium, combined with its high market price, has led to alternatives for performing experimental studies. Therefore, the aim of which is to utilize predictive models, that provide reliable results of the machining process (such as the cutting area temperature, tool wear, cutting forces, chip geometry and surface quality)^4^. However, a good model of the machining process is often difficult to obtain because of the complex nature of the process itself, involving high strains, strain rates and temperatures. The numerical models, most of which are based on the Finite Element Method (FEM), usually are presented as a suitable tool to perform a reliable analysis. This is essential to improve the quality of the machining processes, not only from a functional perspective but also from an economic point of view^10^.

Even though the literature regarding this topic is vast, the simulation models that comprise the constitutive equations, parameters, etc., are constantly changing and improving. This helps to obtain results that better adapt to the real behavior of the alloy under study. Thus, due to the complexity of the material, the existing proposed FEM models are constantly changing based on newly discovered knowledge? Recent articles show that this field of study is still evolving and highlight the mentioned complexity of the material. Some examples can be found in the studies of Chen et al.^11^, where different constitutive models such as the Johnson–Cook (JC), the Johnson–Cook Modified (JCM) and the Khane Huange Liang model (KHL) are compared. In the chip formation simulation model of Ti6Al4V in orthogonal cutting, Maohua et al.^12^ implemented a new analytical formulation for the friction coefficient at the tool-chip-workpiece interface, verifying the results with experimental analysis. Childs et al.^13^ combined FEM simulation with experimental tests to develop a constitutive equation. This combination better describes the flow stress,failure behavior and also examines which parameters are critical to obtain good results. Sadeghifar et al. ^14^, implemented the Johnson–Mehl–Avrami–Kolmogorov (JMAK) recrystallization model. This model is used to study the effect of grain size in Ti6Al4V machining using different cutting parameters. They studied how the surface integrity is linked to the fatigue life and corrosion resistance of machined parts. Bai et al.^10^ have also proposed another analytical model for chip formation prediction in orthogonal cutting of Ti6Al4V.

Even though there are several studies on Ti6Al4V, the material parameters are, in most cases (mainly the flow and failure data), provided by different sources which are assumed to be valid. However, it is uncertain that these obtained parameters match the material behavior for the specific machining process being simulated^13^. It is well known that these material parameters can differ from one article to another, sometimes under the same machining conditions, because there is no specific FEM model in each case or a general model. This might occur when, for example, the JC equation^15^ and the fracture energy (*G*_*f*_) are used to simulate the material fracture behavior. *G*_*f*_ is associated with the material in order to characterize fracture during the cutting process (shear failure criterion^16^). This value can be obtained from previous research, although it does not have a stable value, or through experimental methodology. However, this is time consuming and might not be economically and technically feasible.

So, this study aims to establish a relationship between the cutting parameters and the fracture energy to avoid being dependent on complicated experimental methods or values published in previous studies. This adapts the fracture energy value to the cutting parameters of the selected process. The cutting parameters implemented in machining processes highly influence the material behavior and so, the fracture energy should not be maintained as fixed but adapted. Especially in the machining processes, where extreme conditions are presented throughout the entirety of the cutting process and the material behavior is affected by it. Also, the simulation process can be better adjusted to the material behavior adapting the fracture energy, depending, in this case study, on the cutting parameters. One of the main objectives of the present work is to study the influence of the fracture energy on the machining process for different cutting parameters using FEM and to compare it with experimental results. The parameter *G*_*f*_ has been selected since the JC fracture equation only defines the initiation of the crack. However, *G*_*f*_ governs the damage evolution thus providing results that might fit better to the real behavior of the material. It should also be emphasized that the fracture energy is usually mode dependent and can therefore depend on the loading.

The JC-model and JC damage criterion considers plasticity and damage initiation. This takes into account the strain, strain rate and temperature. The JC damage criterion has been commonly employed in simulation of segmented chip formation when machining titanium alloys like the one considered in this study. The main JC parameters that can be found implemented in the literature do not, in general, present a significant variation^11,17,18^ but *G*_*f*_ can deviate considerably depending on the fracture mode considered (from 11.53 MJ/mm^2^^17^ to 33.67 MJ/mm^2^^19^). This material parameter is obtained from complex experimental tests^20^, taking the direction of the load application among others into account. However, the forces developed during the machining process are complex and so, its direct implementation in the numerical model can be complicated^21^. *G*_*f*_ is considered in general as a constant of the material even though it is affected by the process conditions^22,23^.

It is well-known that an inherent property of this type of failure modeling produces mesh dependent results^24,25^. In the case of JC dynamic failure criterion, where elements are continuously degraded and deleted when the failure criterion is reached, the released energy depends on the element size. When a material point softens in continuum damage models, the softening becomes localized, promoting further softening. The mitigation of damage localization and mesh dependency is outside the scope of the current paper, being focused on the relation of the cutting parameters and the fracture energy. Therefore, the element mesh is kept fixed during simulations and the reported fracture energies are only valid for the specific mesh used in this paper.

The main intention of this study is to analyze the influence of the fracture energy mentioned above using the Finite Element Analysis (FEA) and to establish an empirical relationship between the *G*_*f*_, the cutting parameters and the force at fracture in the machining of the Ti6Al4V titanium alloy. The aim is to avoid being dependent on complicated experimental methods or values published in previous studies, presenting the fracture energy values that better adapt to the conditions simulated and fit the experiments conducted.

## Materials and methods

As mentioned, the present work considers the study of the Ti6Al4V alloy machining process using finite element analysis and experimental tests. An empirical relationship based on the cutting parameters for the simulation model is established and fitted to the results. The empirical equation is also validated by comparing the simulations with the experiments performed for different ranges of cutting parameters.

### Experimental tests

The composition of the selected Ti6Al4V alloy is presented in Table 1 and is obtained by arc atomic emission spectroscopy (AES).

**Table 1:** Machined Alloy composition (mass %).

Alloy	C	Fe	N	O	Al	V	Ti
Ti6Al4V	0.08	0.164	0.05	0.05	5.47	4.09	Rest

The selected cutting parameters for the experimental tests of this alloy^26,27^ (Table 2) have been chosen based on the industrial requirements. A total of 180 tests were performed, with 15 repetitions for each cutting parameter combination to ensure repeatability. The experiments are the same as in previous studies made on this alloy by the authors^28–30^. This facilitates a quick evaluation of the experimental results, reducing experimentation times.

**Table 2:** Cutting parameters for dry turning tests.

*f* (mm/r)	*v*_*c*_ (m/min)	*a*_*p*_ (mm)
0.05	30 65 125	1
0.10		
0.20		
0.30		

The tests were performed in a parallel lathe (ECLIPSE model, ALECOP), equipped with FAGOR 8055 T Numerical Control, in dry conditions. To be able to minimize the geometric variable influence, all tests were carried out in an orthogonal configuration. The specimens were designed with a tailored geometry to maintain orthogonal conditions throughout the tests. Different grooving operations were carried out on a billet (L = 170 mm, D = 105 mm) to achieve a tubular geometry. Then, two crowns were formed, corresponding to the two diameters machined previously. Each crown was machined with a specific thickness equal to cutting depth. Additionally, a relief zone was established, eliminating a sector of the crowns, to ensure that the spindle reached a permanent regime. Therefore, the aim was to obtain results close to a two-dimensional behavior with this geometry and to allow comparisons with the FEM studies carried out, as they are usually implemented to minimize computational time^18,29,31^. A new coated WC–Co insert was used for each test to maintain the same initial conditions. For the monitoring of the cutting forces (*Fc*), a piezoelectric sensor dynamometer, 9121 Kistler with an amplifier and dynamic signal analyzer and a Pulse Labshop data acquisition system were used.

The generated chips were collected, codified, photographed and stored after each test using metallographic techniques for analysis. For the study of the chips, an inverted metallurgical microscope (EPIHOT 280 NIKON, Tokyo, Japan) and a CF Optical System (1.5 × to 400 ×) for the SOM images were used. The corresponding measurements of the samples were obtained using a digital-image-processing software (Omnimet BUEHLER, Lake Bluff, IL, USA). A complete study and representation of the chip geometry is presented in^28^.

The experimental results indicate that a relationship can be obtained between *Fc*, *f*, *v*_*c*_, the height of the peaks (*h*_*p*_) and the height of the valleys (*h*_*v*_). For the validation of the model and the fracture energy equation, the results are compared with the results obtained from the FEM models.

### Finite element analysis

For the Finite Element Analysis, a 2D FEM Lagrangian formulation model is implemented using Abaqus/Explicit. The general JC flow stress model is used for the description of the visco-thermo-plastic behavior of the material [Eq. (1)]. This law considers in a multiplicative way the work hardening, strain rate hardening and thermal softening of the workpiece material at high cutting speeds. The JC law is usually applied in FEM studies of machining including new parameters^32,33^ or not^11,12^. Equation (1) shows the JC law, where $\sigma$ is the flow stress, $\varepsilon$ is the equivalent plastic strain (representing the work hardening based on the constants A, B and n), $\dot{\varepsilon }$ the strain rate, ${\dot{\varepsilon }}_{0}$ the reference strain rate (representing the strain rate based on the constant C), ${T}_{r}$ the ambient reference temperature and ${T}_{m}$ the melting temperature of Ti6Al4V (representing the thermal effect based on the constant m). The parameter A is the initial yield strength of the material at quasi-static strain rate. The parameter B and n represents the flow stress on strain hardening behavior at quasi-static strain rate. And the parameter C represents strain rate effect, and m represents thermal softening effect. Table 3 shows the JC model parameters implemented for this study^11^. The reference strain is defined as 0.7 s^−1^^17^.

**Table 3:** JC model parameters for Ti6Al4V.

*A *(MPa)	*B *(MPa)	*n*	*C*	*m*	*Tr* (K)	*Tm *(K)
870	990	0.25	0.011	1	298	1933

1 $$\sigma =\left[{\mathrm{A}}+{\mathrm{B}}{\left(\varepsilon \right)}^{\mathrm{n}}\right]\left[1+{\mathrm{C}}ln\left(\frac{\dot{\varepsilon }}{{\dot{\varepsilon }}_{0}}\right) \right]\left[1-{\left(\frac{T-{T}_{r}}{{T}_{m}-{T}_{r}}\right)}^{\mathrm{m}}\right]$$

Also, for the damage initiation, the JC fracture model or damage criterion^12,34^, given in Eq. (2), is used to define the initial failure strain. Table 4 shows the model parameters, where ${\varepsilon }_{oi}^{-pl}$ is the equivalent plastic strain at the onset of damage (assumed to be dependent on ${\dot{\varepsilon }}^{-pl}/{\dot{\varepsilon }}_{0}$ a non-dimensional plastic strain rate, $p/q$ a dimensionless pressure-deviatoric stress ratio (being $p$ the pressure stress and $q$ the von Mises stress), ${T}^{*}$ the non-dimensional temperature defined previously in Eq. (1) $(T-{T}_{r}/{T}_{m}-{T}_{r}$ and ${\mathrm{d}}_{i}$ are the failure parameters^11^.

**Table 4:** JC Damage model parameters for Ti6Al4V.

${\mathrm{d}}_{1}$	${\mathrm{d}}_{2}$	${\mathrm{d}}_{3}$	${\mathrm{d}}_{4}$	${\mathrm{d}}_{5}$
− 0.09	0.25	− 0.5	0.014	3.87

2 $${\varepsilon }_{oi}^{-pl}=\left[{\mathrm{d}}_{1}+{\mathrm{d}}_{2}exp\left({d}_{3}\frac{p}{q}\right)\right]\left[1+{\mathrm{d}}_{4}ln \left(\frac{{\dot{\varepsilon }}^{pl}}{{\dot{\varepsilon }}_{0}}\right) \right]\left[1-{\mathrm{d}}_{5}\left({T}^{*}\right)\right]$$

The criterion for the damage evolution is based on the fracture energy (*G*_*f*_) with exponential softening [Eq. (3)].

3 $$D=1-exp\left({\int }_{0}^{{u}_{p}}\frac{\tilde{\sigma }}{{G}_{f}}d{u}_{p}\right)$$

where *D* is the damage, $\tilde{\sigma }$ the effective stress and ${u}_{p}$ the equivalent plastic displacement. When the damage progresses in a specific element and reaches *D* = 1, the element is considered fully damaged and is consequently removed from the analysis.

The element deletion is activated, so the elements that reach the distortion marked for the simulation (*G*_*f*_) are eliminated from the simulation.

For the contact between the chip and the cutting tool, a surface-to-surface contact definition is applied. A penalty friction formulation based on Zorev’s model [Eq. (4)] is used to define the tangential behavior^35^

4 $$\tau =\left\{ \begin{array}{c} \mu {N}_{f}, \tau <{\tau }_{limit} (sliding)\\ {\tau }_{limit}, \tau \ge {\tau }_{limit} (sticking)\end{array}\right.$$

where *τ* the frictional stress, *µ* the apparent friction coefficient at the cutting tool/chip interface, *µN*_*f*_ the friction along the contact length and *τ*_*limit*_ the material shear stress, remaining constant *µ* and *τ*_*limit*_ (*µ* = 0.3 and *τ*_*limit*_ = 331 MPa). The normal behavior is defined as a hard contact. However, the friction variable has been set based on previous analysis and it is not part of this analysis^36,37^. The heat transfer coefficient at the interface was modelled using a high thermal conductance in order to reach the steady state quickly.

**Figure 1:**
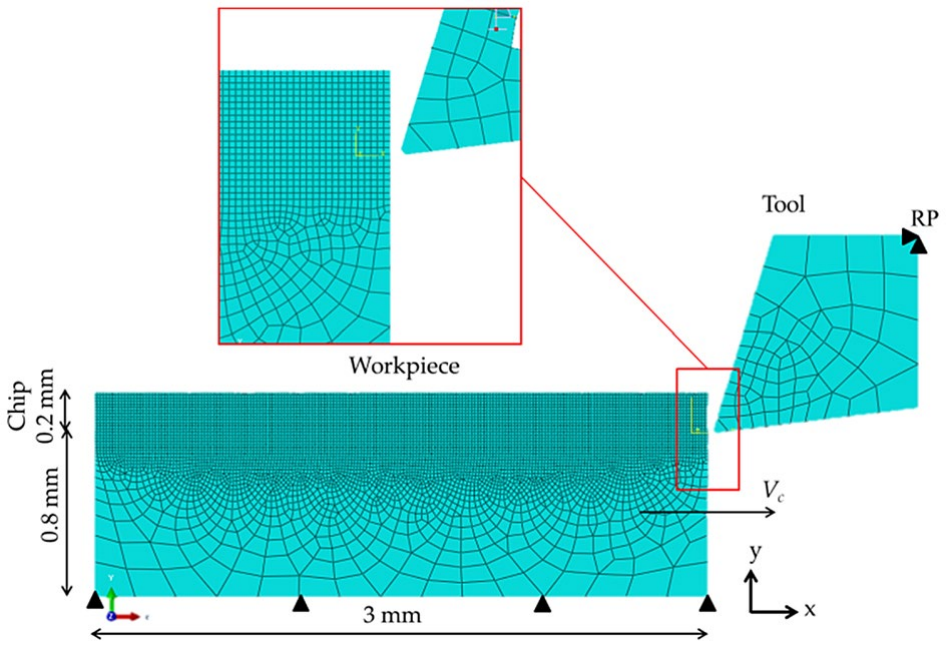
FEM model, mesh and boundaries.

Figure 1 shows the mechanical boundary conditions for the model. As illustrated, the displacement is set to zero in the y-direction at the bottom of the workpiece. In addition, the cutting speed is applied in the x-direction at the bottom of the workpiece. The cutting tool is constrained to a Reference Point (RP) and is set to zero. With regard to the thermal boundary conditions, a starting temperature of 298 K is applied to the workpiece and the tool in the initial step.

According to the consulted literature, the mechanical and thermodynamic parameters for the workpiece and the tool are as presented in Table 5^32,38^.

**Table 5:** Ti6Al4V and tool parameters used in the FEM simulations.

**Mechanical and thermal properties for Ti6Al4V**	
Density (t/mm^3^)	4.5E−09
Young’s modulus (MPa)	144,000
Poisson’s ratio	0.32
Expansion coefficient (m/m K)	9.40E−06
Specific heat (J/kg)	656
Thermal conductivity (W/m K)	6.6
Melting point (K)	1933
**Thermal and mechanical properties of the cutting tool**	
Density (t/mm^3^)	1.19E−08
Young’s modulus (MPa)	–
Poisson’s ratio	
Expansion coefficient (m/m K)	
Specific heat (J/kg)	33,700,000
Thermal conductivity (W/m K)	86

For decreasing the computational time, the mesh of the workpiece is divided into two different domains (Fig. 1). The upper part, which is subjected to severe deformation due to the machining process, is meshed with smaller elements. The rest of the workpiece is meshed with larger elements further away from the cutting area. The tool is divided into two areas; the first one around the contact zone with the chip and the second far away from the cutting area. The element size and shape, as well as the meshing control for each area, are shown in Table 6. As for the boundary conditions, the bottom of the workpiece has been fixed and no movement is allowed in the x-direction or y-direction. As for the cutting tool, a reference point (RP, Fig. 1) has been placed on the upper part to be able to include the cutting speed in the *x* direction.

**Table 6:** Meshing features.

	Area	Element size (mm)	Element shape	Meshing control
Workpiece	Chip	0.015	Quad	Structured
	Rest	0.015–0.3	Quad-dominated	Free
Tool	Cutting	0.06	Quad-dominated	Free
	Rest	0.06–0.20	Quad-dominated	Free

The general element type applied for the workpiece and cutting tool is CPE4RT (4-node plane strain thermally coupled quadrilateral, bilinear displacement and temperature, reduced integration, hourglass control). For a better transition between the areas with different elements size, the element type CPE3T (3-node plane strain thermally coupled triangle, linear displacement and temperature) is used. The model has a total of 6514 number of elements and 6639 number of nodes.

Also, in order to complete the simulations and consume as little computational time as possible, it is important to avoid severe element distortion as much as possible. Therefore, it is essential to create a suitable mesh. As mentioned above, it is also well-known that the results are mesh dependent for materials which exhibit softening^19,39,40^. The mesh dependency is unavoidable and therefore it is referred to as model data instead of true material data. However, using the same mesh configuration, size, elements, etc. with no remeshing, it is assumed that the results are valid for this model. An optimization study has been conducted to set the final mesh used in all the simulations.

## Results and discussion

To obtain the empirical relation, a set of simulations are carried out using a set of *G*_*f*_ values. In the literature, a fracture energy of 33.67 mJ/mm^2^ is commonly used for this alloy^17^. However, it is not possible to maintain this value for the set of cutting parameters, because of the differences between the simulation and the experimental results, but also due to the simulation problems encountered with mesh distortion etc.

Having this parameter fixed for the wide range of cutting parameters simulated is also inadequate. This is mainly due to its dependency on the changes in the fracture mechanics of the cutting process. A *G*_*f*_ adaptation for a low or a high speed, as well as for the different feeds, provides more suitable results, as can be seen in this section.

**Figure 2:**
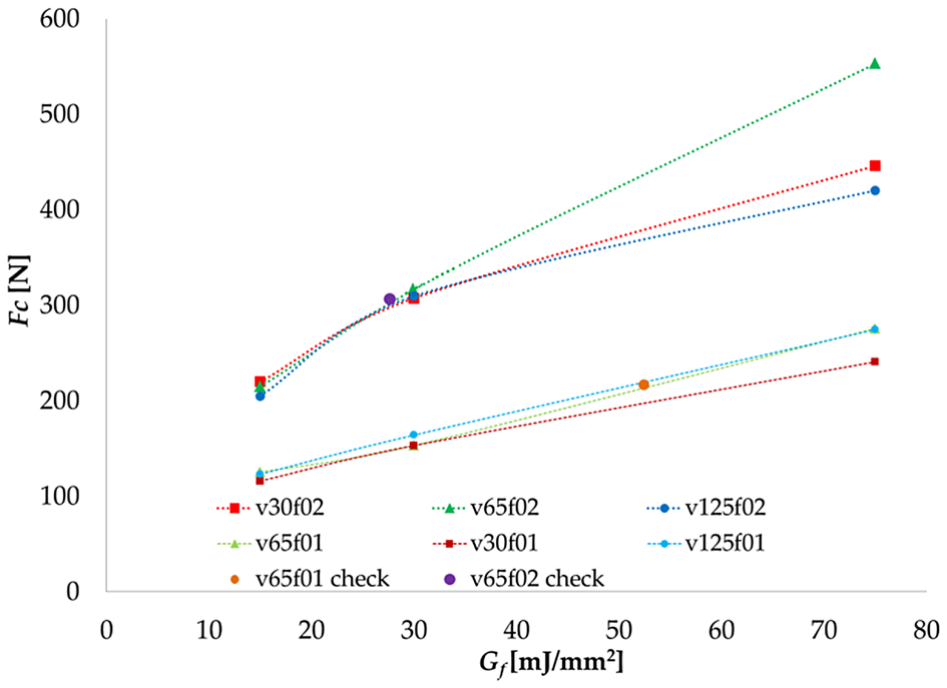
Cutting force evolution based on the fracture energy.

Several simulations are performed using different fracture energies to obtain the dependence of the cutting forces on the feed, cutting speed and *G*_*f*_. Due to the computational time requirements, the simulations are limited to two different feeds, as seen in Fig. 2. Also, in Table 7, the simulated chips are shown. Depending on the applied *G*_*f*_ different behavior can be observed. All the parameters have been fixed except for *G*_*f*_ and the feed.

**Table 7:** Chip evolution depending on the *G*_*f*_ applied for 65 m/min.

*f* (mm/r)	*G*_*f*_ (mJ/mm^2^)		
	15	30	75
0.1			
0.2			

Based on the cutting forces provided from the experimental results in^28^ and presented in Table 8, the fracture energy is obtained from Fig. 2 using the corresponding cutting speed and feed. The empirical relation [Eq. (5)] is then obtained by applying a multivariable linear regression to determine the unknown parameters, presenting a R^2^ of 0.91. By studying the results obtained from the experiments and the FEM model, the empirical relation for *G*_*f*_, the cutting parameters and the cutting force has been obtained [Eq. (5)]. This can be used to determine *G*_*f*_ from experiments by measuring the cutting force from the experiments, using the corresponding feed and cutting speed. The final equation is given by

**Table 8:** Cutting parameters for dry turning tests.

*v*_*c*_ (m/min)	*f* (mm/r)	*Fc* (N)	*G*_*f*_ (mJ/mm^2^)
30	0.10	255.75	88.50
	0.20	294.23	29.69
65	0.10	209.92	54.85
	0.20	309.29	30.32
125	0.10	75.93	34.05
	0.20	154.44	15.40

5 $${G}_{f}=0.2253\cdot {v}_{c}^{-0.3132}\cdot {f}^{-1.3161}\cdot {F}_{c}^{0.6973}$$

Two more simulations are made with the predicted *G*_*f*_ for a cutting speed of 65 m/min and a feed of 0.10 and 0.20 mm/r in order to confirm the results obtained from Eq. (5). The resulting cutting forces from each simulation give a good fit to the fracture energy given by Eq. (5), as it can be seen in Fig. 2 (v65f01 check and v65f02 check).

Once the equation is assumed to be valid, a set of simulations are performed using the cutting parameters in Table 2. From the obtained results, the cutting forces and the chip geometry are compared with the ones acquired from the experimental tests.

**Figure 3:**
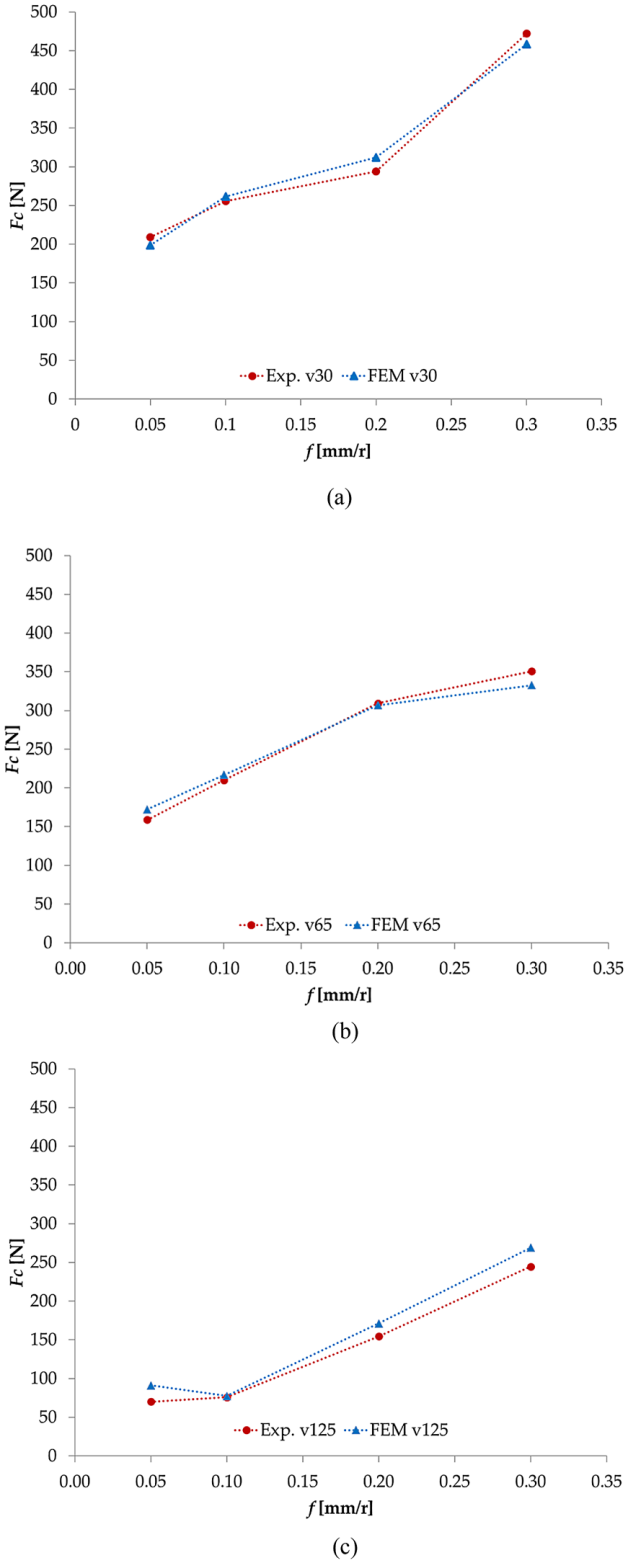
Cutting forces comparison between experimental results and FEM for 30 (**a**), 65 (**b**) and 125 (**c**) m/min.

Figure 3 shows the comparison of the cutting forces for the implemented range of cutting parameters. It can be seen that the empirical relation works good for the implemented feed range. Table 9 shows the calculated *G*_*f*_ and the simulated and experimental cutting forces and the corresponding error for each couple of cutting parameters. It can be seen that the error is in general below 10%. Only for a 125 m/min and 0.05 mm/r, the simulation result error is over 10%, which can be explained due to the mechanical behavior of the Ti6Al4V at a higher cutting speed. This type of alloy presents a low thermal conductivity which leads to a rapid increase in temperature in the primary deformation zone, having an adiabatic process. So, the material softens and it is easily deformed^29,41^. Also, as *v*_*c*_ increases, friction is reduced and so the cutting forces are lower^42^. These kinds of mechanisms are not included in such an empirical relation as the one presented in Eq. (5). However, the main objective of this analysis is to work with a simple equation that can be used to determine the fracture energy from cutting forces and cutting data. Knowing its limitations, it is presented as a first approximation.

**Table 9:** Cutting forces evaluation for each *G*_*f*_ calculated.

*Vc* (m/min)	*f* (mm/r)	*Fc* (N) experimental	*Fc* (N) simulation	*Fc* (%)	*G*_*f*_ (mJ/mm^2^) empirical
30	0.05	208.94	198.56	− 5.22	166.02
	0.10	255.75	261.52	2.21	76.77
	0.20	294.23	312.21	5.75	34.00
	0.30	472.05	458.62	− 2.92	27.72
65	0.05	158.90	172.35	7.80	107.67
	0.10	209.92	216.84	3.19	52.51
	0.20	309.29	306.62	− 0.87	27.63
	0.30	350.54	332.47	− 5.43	17.68
125	0.05	70.05	91.06	23.07	49.56
	0.10	75.93	77.43	1.94	21.05
	0.20	154.44	171.23	9.81	13.87
	0.30	244.68	269.08	9.07	11.21

**Figure 4:**
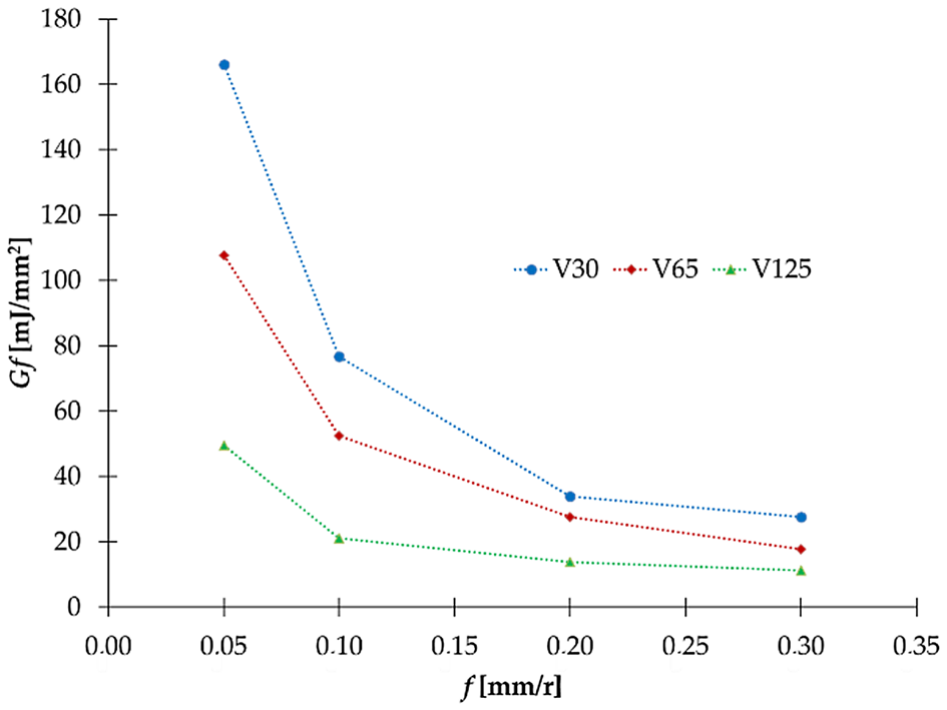
Evolution of the fracture energy as a function of *f* and *v.*

In Fig. 4, *G*_*f*_ is presented as a function of feed and cutting speed. It can be clearly observed that the fracture energy tends to converge to a specific value at a higher feed. This tendency also applies for higher speeds. This demonstrates that less fracture energy is needed to fracture the material at higher values of feed and cutting speed. So, Eq. (5) shows how it is necessary to adapt *Gf* depending on the cutting data choice. This implies the importance of having a mixed-mode *Gf* model due to the complex interaction of loading, geometry etc. as the fracture mode varies in the machining process depending on these parameters^43^.

In order to illustrate the obtained results more clearly, a geometry comparison of the chip formation process and the experimental results^28^ has been carried out. The results of the study are presented in Table 10. It can be seen that the cutting forces are in good agreement with the experimental results. The model presents similar chip formation for higher range of feeds (0.30–0.20 mm/r). However, it is not possible to capture the serrated geometry for the lower range of feed (0.10–0.05 mm/r). This might be explained due to the changes in the fracture mechanism. For higher *f*, the cutting process develops higher shear forces that can be well represented with the JC formulation implemented. However, for lower *f*, the portion of material that is submitted under the cutting tool thrust is less (thinner). Because of this, the normal force is bigger than the shear force and the model is not able to present the characteristic serrated geometry. It can also be observed that the mesh consists of fewer elements in the chip zone resulting in a stiffer behavior. This can be improved with a thinner mesh but, as explained in previous paragraphs, a thinner mesh, brings more elements and that more time is needed for the simulation. So, for this first approximation, the mesh is maintained.

**Table 10:** Chip geometry comparative between experimental tests and FEM model.

*Vc* (m/min)	*f* (mm/r)			
	0.30	0.20	0.10	0.05
30				
			
65				
			
125				
			

JC material model in simulations can lead to continuous chip geometry for low *v*_*c*_ and *f*^17,44^ but is one of the criteria generally used to simulate the crack initiation and propagation into the primary shear zone during the chip formation. The literature shows several numerical models developed but none of which are adequately adapted to predict the chip formation profile which highlights the difficulty behind the replication of this mechanism and the existing gap^4^. Also, the chip formation mechanism of Ti6al4V is still not well understood due to the complexity of the material behavior itself^42^. It can be seen in the literature, that some authors consider that chip formation in this kind of alloy is due to a thermoplastic instability, while others consider the initiation and propagation of cracks inside the primary shear zone of the workpiece material. Moreover, the microstructural state of the alloy strongly influences the chip formation. This alloy presents a transitional chip at lower *v*_*c*_, showing a more segmented chip at higher *v*_*c*_^41^, as it shows in the experimental tests.

As stated in previous research^28^, over the experimental tests performed the serrated chip morphology remains continuous across the *v*_*c*_ and *f* range studied, due to the high plasticity levels of this alloy and its low thermal conductivity, resulting in thermal softening, making the chip more difficult to break. This kind of behavior and the characteristics explained in previous paragraphs are what make it difficult to translate the alloy behavior to the simulation experiments.

Taking into account this brief explanation, it can be understood that the simplicity of the model is not taking into consideration all the parameters that influence the chip formation for this alloy. However, this first approximation of the model fits well to high feeds but not to low feeds. Therefore, the numerical model can be improved in this aspect. It can be identified in the simulations that, for the lower *f* of 0.05 mm/r, the chip tends to have a serrated shape with an increase in *v*_*c*_. For 125 m/min and 0.05 mm/r, the chip has a more pronounced wavy shape than for lower *v*_*c*_, but does not create a serrated shape. The same happens, less obvious, for *f* = 0.10 mm/r. In this case, the wavy effect disappears. This effect depends, in this case, on the mesh implemented. For *f* = 0.10 mm/r it seems that there are not enough elements to appropriately represent the chip shape. For *f* = 0.05 mm/r, the distortion of the mesh is such that some spaces and jumps between elements appear. This behavior, although is not well represented, follows the behavior presented by the alloy during the experimental tests. In the lowest feed rate range (0.05–0.10 mm/r), the chip was continuously helical and showed a tendency to create chip nests for 0.05 mm/r. This could be understood with the less serrated morphology of the chip obtained during simulation.

**Figure 5:**
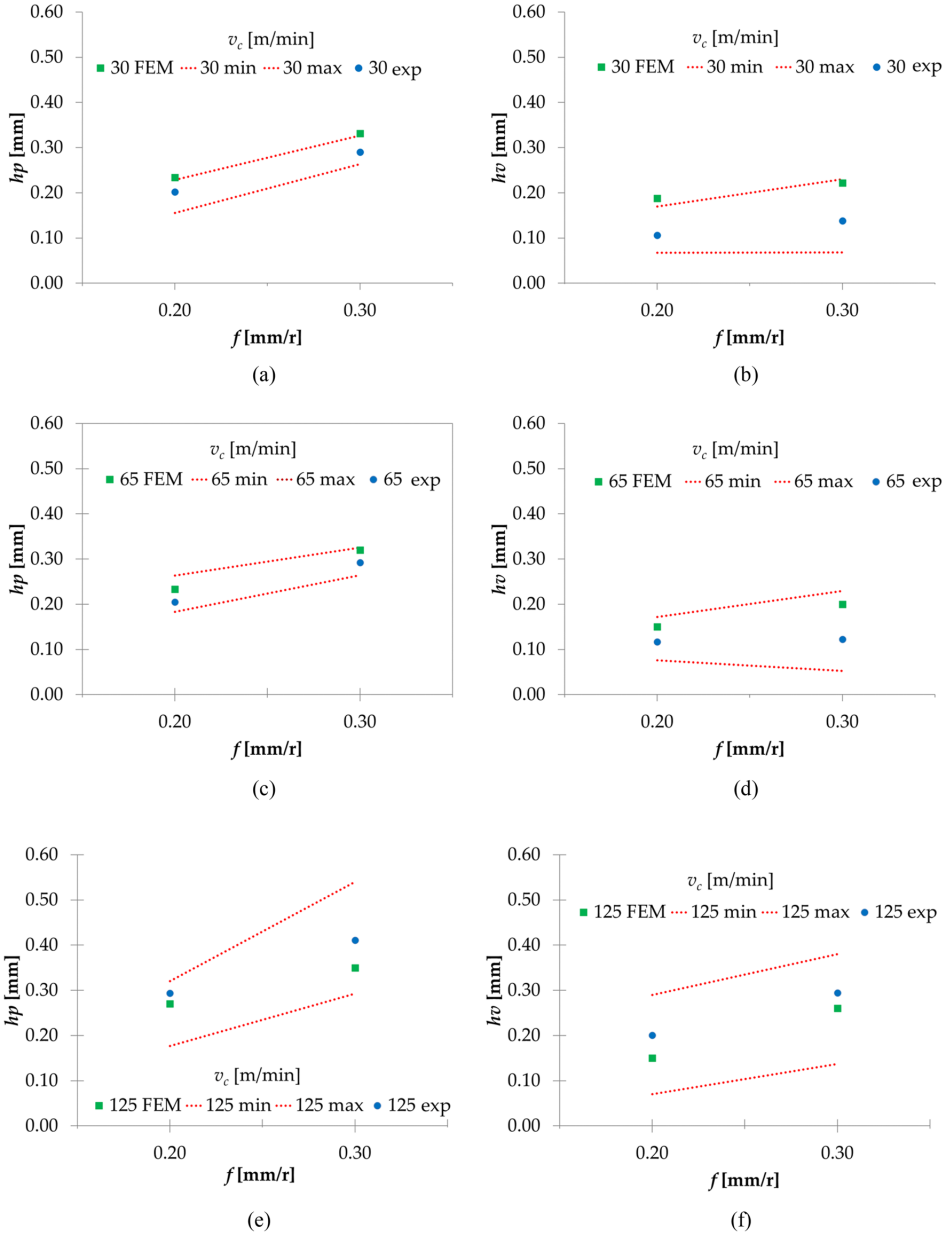
Evolution of *h*_*p*_ and *h*_*v*_ for the experimental and FEM tests.

The geometrical data obtained from the experimental tests are shown in Table 11^28^ and the comparison between the average *h*_*p*_ and *h*_*v*_ is presented in Fig. 5 and Table 12. This is only shown for 0.20 and 0.30 mm/r because, as shown in Table 10, for the other *f*, the chip geometry does not present a serrated shape. The average results of *h*_*p*_ and *h*_*v*_ present a larger error compared with the ones obtained from the experimental tests (Table 12). Considering the spread in the experimental measurements (maximum and minimum experimental values, as shown in Fig. 5 as “min” and “max”) and not only the average value (“exp”), but it can also be observed that the results obtained from the simulation are in general within the range of the experimental results. The maximum error is 43.5% for *v*_*c*_ = 30 m/min and *f* = 0.20 mm/r and the minimum is 8.5% for a *v*_*c*_ = 60 m/min and a *f* = 0.30 mm/r. It can be estimated that with an increment of the *v*_*c*_ and the *f*, the error % is reduced, except for 125 m/min, where the trend changes. This can be attributed to the same mechanism explained in relation to Table 10. As an average the model presents an error of 12% and for the valley values and 30% for the peak values.

**Table 11:** Chip geometrical average data, being *hp* the heights of peaks, *hv* the heights of valleys, *tc* the equivalent chip thickness and *S* the segment width in mm.

	Vc (m/min)											
	30				65				125			
	h_p_	h_v_	t_c_	S	h_p_	h_v_	t_c_	S	h_p_	h_v_	t_c_	S
**f (mm/r)**												
0.05	0.054	0.044	0.06	0.014	0.050	0.021	0.059	0.01	0.040	0.025	0.061	0.02
0.1	0.095	0.072	0.114	0.029	0.110	0.074	0.122	0.05	0.100	0.055	0.138	0.04
0.2	0.202	0.106	0.264	0.116	0.200	0.117	0.261	0.09	0.190	0.100	0.247	0.09
0.3	0.290	0.138	0.365	0.116	0.290	0.122	0.361	0.13	0.320	0.175	0.353	0.15

**Table 12:** Model error percentage for the average *h*_*p*_ and *h*_*v*_ values compared with experimental results.

*v*_*c*_ (m/min)	*f* (mm/r)	Error (%)	
		*h*_*p*_	*h*_*v*_
30.00	0.20	13.60	43.45
	0.30	12.49	37.79
65.00	0.20	12.22	22.08
	0.30	8.59	38.76
125.00	0.20	− 8.88	− 33.83
	0.30	− 17.41	− 13.35

It can be concluded that the initial model provides reasonably good fit to the experimental results regarding the obtained cutting forces. For the chip geometry analysis, the model can be valid for a feed range between 0.20 and 0.30 mm/r. Thus, it is necessary to develop the model further to better adapt to the low feed range (0.05 and 0.10 mm/r).

As a model control, the energy balance, which must be identified, quantified and minimized to prevent erroneous findings, is analyzed, presenting a 5% of error, intended to be improved in future work. To control that the numerical model adhere to the basic physical laws (conservation of energy), the global energy of the model is checked to analyze that there are no major inconsistencies in the energy of the system, e.g. keeping the sum of internal, kinetic, sliding, hourglass, system damping, and rigid wall energies within an acceptable range. According to the literature consulted, this acceptable range should be around a 5% of the total global energy^45,46^.

Nevertheless, the developed model is presented as a preliminary study for the range of values studied. It would require a more in-depth study to analyse and incorporate the fracture mechanism mentioned, by which the chip generation can be closer to reality. A mixed-mode fracture criterion with different fracture energies in tension/peel and shear should be implemented and tested to further evaluate the model.

## Conclusions

In this work, a 2D FEM model for the analysis of the machining process of the Ti6Al4V alloy with adapted fracture energy values for each cutting parameter set has been developed. Additionally, an empirical equation to obtain the optimum fracture energy is presented. This involves maintaining the simulation parameters fixed and adapting *G*_*f*_ depending on the cutting parameters. A validation of the FEM model with experimental results obtained in previous studies has been made in order to establish the methodology and select the optimal values. This shows the advantages and disadvantages of the analysis. For this comparison, the *Fc*, *h*_*v*_ and *h*_*p*_ are selected to analyze the model mechanical and geometrically.

Implementing the *G*_*f*_ obtained from the empirical equation developed, the simulation model shows results in good agreement with the experimental tests. The main comparison is made with the cutting forces, presenting a value error lower than 10% for most of the cutting parameters studied. For a *v*_*c*_ of 125 m/min the data evolution presents a higher error, but only for a *f* of 0.05 mm/r this percentage is over 10%. This is due to the mechanical behavior of the Ti6Al4V at higher *v*_*c*_ (low thermal conductivity, high chemical reactivity and high hardening) and the simplicity of the equation.

As for the chip geometry, the model is able to represent the serrated characteristic chip for high *f* (0.30 and 0.20 mm/r). However, this shows an average error between peaks and valleys of 12% and 30% correspondingly. For lower *f*, the model is not able to obtain a serrated chip, due, again, to the simplicity of the initial model. The fracture mechanism changes depending on the *f* implemented and the model is not developed to implement these variables in the first stages of the study.

This work attempts to highlight the necessity of adapting the fracture energy to the machining process parameters during FEM simulations, due to the influence that this parameter has upon the mechanical behavior of the material being machined. The aim is not to obtain the value from previous literature works or complicated experimental methods, which hardly represent the mechanism behind the machining process analyzed.

It is necessary to point out that this work is only the first stage of the cutting forces and chip morphology analysis of the Ti6Al4V alloy by FEM. The study and results are established for the specified cutting conditions but with an appreciable adjustment to the experimental values presented. Other mechanical and geometrical parameters, such as the temperature, shear stresses, chip segmentation ratio or shear angle, will be addressed in further works. In addition, the generality of the empirical equation should be tested in a wider range of cutting speed and cutting depth.
